# Prevalence of positive TST among healthcare workers in high-burden TB setting in Peru

**DOI:** 10.1186/s12889-020-08756-9

**Published:** 2020-05-03

**Authors:** Juana Sedamano, Alvaro Schwalb, Rodrigo Cachay, Carlos Zamudio, César Ugarte-Gil, Gabriela Soto-Cabezas, César V. Munayco, Carlos Seas

**Affiliations:** 1 Instituto de Medicina Tropical Alexander von Humboldt, Universidad Peruana Cayetano Heredia, Lima, Peru; 2 Facultad de Medicina, Universidad Peruana Cayetano Heredia, Lima, Peru; 3 Centro Nacional de Epidemiología Prevención y Control de Enfermedades, Ministerio de Salud, Lima, Peru

**Keywords:** Tuberculin test, Healthcare workers, Latent tuberculosis infection

## Abstract

**Background:**

Tuberculosis (TB) transmission has long been recognized as an important occupational hazard for healthcare workers (HCWs). HCWs have a 5.8% annual risk of exposure and three times greater risk of developing active TB than the general population.

**Methods:**

We conducted an observational cross-sectional study between September 2014 and March 2015 among HCWs in a high-burden TB setting in Lima to estimate the prevalence of positive Tuberculin Skin Test (TST) and to investigate factors associated with a positive TST.

**Results:**

Two hundred forty participants were included in the analysis; TST was administered to 190 (79.2%) while the rest were exempt due to a previous positive TST result, history of TB, or test refusal. A positive TST result was found among 56.2% of participants to whom the TST was applied (95% CI: 49.22–63.55%). When considering those who had a previous positive TST result and those with a history of TB, the prevalence of a positive TST result was 64.3% (95% CI: 57.8–70.3%). No significant differences were observed between clinical/paramedical and administrative staff in the health center. The use of N95 masks during work hours was reported by 142 (69.9%) participants. Prevalence ratios (PR) show that workers with more than 120 months as a HCW were 1.44 times more likely to be TST positive. The multivariate analysis found that HCWs with over 10 years of service were 1.52 times more likely to be TST positive.

**Conclusion:**

This study supports previous reports that TB infection is an occupational hazard for HCWs. Prevention of TB transmission through control measures, as well as timely diagnosis of LTBI in this particular high-risk group, is critical for individual and public health.

## Background

According to the World Health Organization (WHO), approximately one-quarter of the world’s population is estimated to have latent tuberculosis infection (LTBI) [1]. LTBI constitutes a major challenge for tuberculosis (TB) control due the fact that it incurs a lifetime risk of developing active TB disease. It has been estimated that 5–10% of LTBI cases will progress to active TB, typically within the first 5 years after the initial infection [2]. LTBI can be effectively treated to reduce the risk of progression to active TB by 60–90% [3].

TB transmission has long been recognized as an important occupational hazard for healthcare workers (HCWs), with a 5.8% median annual risk attributable to TB exposure [4, 5]. WHO guidelines recommend systematic screening for TB in at-risk populations including HCWs, prisoners, and immigrants from high-burden countries [3]. Traditionally, tuberculin skin testing (TST) has been widely used as a cheap diagnostic test for LTBI, but recently, interferon-gamma release assays (IGRA) have emerged as an alternative that provides greater specificity, though at a higher cost [6]. A recent systematic review estimated the prevalence of LTBI (measured by TST) in HCWs to be 51% [7], while another concluded that, when compared to the general population, the risk of LTBI was 2.27 times greater for HCWs [8].

In 2015, around 31,000 new cases of TB were registered in Peru; Lima and Callao notified 59.3% of these cases [9]. Therefore, HCWs working in these areas are exposed to more than half of the country’s TB burden. Previous studies of LTBI prevalence in Callao have revealed IGRA-positive results in 56% of HCWs [10]. Such studies have not been performed in San Juan de Lurigancho (SJL), a district of Lima with an extremely high TB prevalence compared to other districts [11]**.** The main objective of this study was to determine the prevalence of LTBI via TST in HCWs at SJL health centers and to identify factors associated with a positive TST result.

## Methods

### Study design

We conducted a cross-sectional observational study between September 2014 and March 2015 in SJL health centers, which provide primary care for almost one million people in the district.

### Setting and participants

At present, no studies have addressed community LTBI prevalence in Peru despite the high prevalence of active TB in Peru (approximately 120 new cases per 100,000 inhabitants have been reported in recent years [1]) compared to other Latin American countries; in the last years, approximately 120 new cases per 100,000 inhabitants have been reported [1]. At the same time, the incidence rate of active TB among HCWs decreased from 215 cases in 2011 to 126 cases in 2015 [9]. There are approximately 660 HCWs distributed throughout 34 health centers in SJL; the study population included workers from 13 of these health centers. HCWs were defined as paid workers employed by an institution whose primary intent is to improve health [12]; they were required to be 18 years of age or older and to have been employed by the health center for at least 3 months. Participants were enrolled through convenience sampling.

Tuberculin for TST administration were provided by the Peruvian Center for Disease Control and Prevention for TB surveillance and research in SJL. All participants were interviewed before TST administration using a questionnaire developed for this study **(**Additional file 1**)**; TST was not applied to workers who reported a history of TB, a previous positive TST, or declined the procedure. A case report form included information on demographic and occupational characteristics, history of TB, previous TST results, risk factors for TB, comorbidities including HIV, and previous screening procedures for active and latent TB infection.

### Sample size

We used an error margin of 0.05 and an LTBI prevalence of 54% among HCWs to calculate an appropriate sample size [13]. The result of this calculation was a sample size of 243 participants (95%CI, 49–59) for the total population of 660 HCWs in SJL.

### TST procedure

We performed a single-step TST using 0.1 ml [5 international units (IU)] tuberculin (Tubersol®), administered using the Mantoux method [14]. Research staff read skin reactions 48 to 72 h after TST placement. Self-reporting of results was not allowed. We considered a TST induration size of ≥10 mm in HIV-uninfected persons and a TST reaction of ≥5 mm in HIV-infected persons as positive [15]. All TST-positive HCWs were advised to be vigilant about the development of any active TB symptoms.

### Statistical analyses

We entered data into an electronic database on Excel XP (Microsoft, US) and performed a descriptive analysis with frequencies and percentages. We divided participants into two groups: TST-positive and TST-negative. TST-positive included participants with a history of TB, a previous positive TST result, or a positive TST result during this study; TST-negative included participants with a negative TST result. We performed a bivariate analysis using Poisson regression with robust variance to calculate prevalence ratio (PR); we executed the multivariate analysis using variables from the bivariate analysis that were significant (*p* < 0.05) or known factors such as having a household TB contact, being overweight, and not using N95 masks. Analyses were carried out in Stata SE 15.1 (StataCorp, US).

## Results

**Fig. 1:**
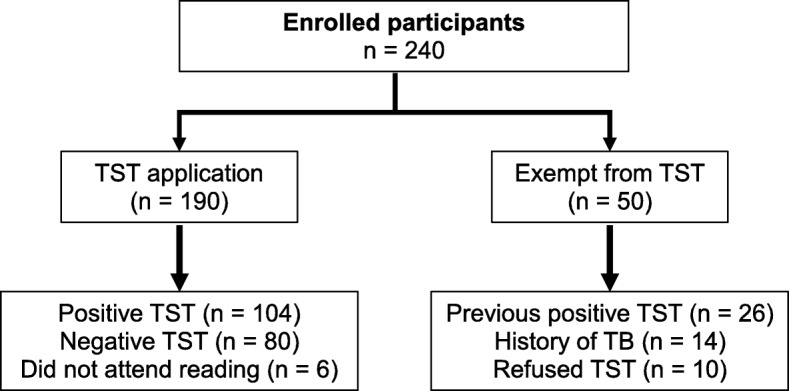
Flow diagram of participants in the study

We enrolled 240 participants in the study, and all completed the interview. Among the 190 (79.2%) participants who were applied the TST, six did not return for the TST-result reading appointment (Response rate: 96.8%). The remaining 50 (20.8%) participants were exempt from TST application because 26 (10.8%) had a previous positive TST result, 14 (5.8%) had a history of TB, and 10 (4.2%) refused TST application **(**Fig. 1**).**

Most of the participants were female (80.8%) and the average age was 41.9 years (Min: 21, Max: 68). The median time working as a HCW was 120 months (IQR: 48–240) and working in the health center was 48 months (IQR: 17–168). Most participants were employed as clinical staff (47.1%), followed by administrative staff (20.4%) and paramedical staff (13.7%) (Table 1).

**Table 1:** Demographic characteristics of HCWs

Characteristic (*n* = 240)	
Age, years, mean (SD)	41.9 (11.2)
Female gender	194 (80.8)
BMI, kg/m^2^, median (IQR)	25.9 (5)
District of residence	
SJL	135 (56.3)
Others	105 (43.8)
Time of residency on SJL, years, median (IQR)	25 (21.7)
Time working as HCW, months, median (IQR)	120 (192)
Time working on Health Center, months, median (IQR)	48 (151.5)
Occupation	
Clinical staff	
Physician	22 (9.2)
Nurse	31 (12.9)
Nursing technician	48 (20)
Obstetrician	12 (5)
Paramedical staff	
Laboratory technician	17 (7.1)
Psychologist	11 (4.6)
Nutritionist	5 (2.1)
Support staff	
Social worker	6 (2.5)
Administrative staff	49 (20.4)
Other staff	39 (16.2)
Underlying disease	
HIV	1 (0.4)
DM	9 (3.8)
HTN	14 (5.8)
Asthma	9 (3.7)
Gastritis	8 (3.3)
Cancer	3 (1.2)
Other	25 (10.4)

Values are n (%) unless noted otherwise

*HCW* Healthcare worker, *SD* Standard deviation, *BMI* Body mass index, *IQR* Interquartile range, *SJL* San Juan de Lurigancho, *HIV* Human immunodeficiency virus, *DM* Diabetes mellitus, *HTN* Hypertension

Regarding biosafety, 205 (85.4%) HCWs provided treatment and care to TB patients. 142 (69.9%) participants reported using N95 masks during work hours; however, only 48 (31.4%) reported use it all the time (Table 2). Concerning active tuberculosis screening, we found that routine sputum sampling and chest radiography was performed in 99 (41.3%) and 128 (53.3%) participants, respectively. 59 (24.6%) participants presented with cough for more than 2 weeks at some point within 1 year prior to the data collection, and the resulting actions taken are described in Table 3. Half of our participants (124) reported having had a TST during the last year. Most participants (85.2%) who were applied the TST agreed to take Isoniazid preventive therapy (IPT) if the result was positive.

**Table 2:** Tuberculosis exposure and workplace biosafety

Characteristic (*n* = 240)	
Directly attend patients	
Yes	205 (85.4)
Use of N95 mask (*n* = 203)	
Always	44 (21.7)
Almost always	49 (24.1)
Sometimes	47 (23.2)
Never	63 (31.0)
Reason for not using N95 mask (*n* = 63)	
They are uncomfortable	11 (17.4)
Masks not provided by health center	38 (60.3)
Does not want to use it	1 (1.6)
Other	15 (23.8)
TB household contact (*n* = 72)	
Yes	22 (30.5)
Received IPT because of TB household contact (*n* = 22)	
Yes	5 (22.7)

Values are n (%)

*TB* Tuberculosis, *IPT* Isoniazid preventive therapy

**Table 3:** Active and latent tuberculosis screening among HCWs

Characteristic (*n* = 240)	
Routine sputum sample during the last year	
Yes	99 (41.3)
Chest radiography during the last year	
Yes	128 (53.3)
Routine TST during the last year	
Yes	124 (51.7)
Cough for more than 2 weeks during the last year	
Yes	59 (24.6)
Action taken (*n* = 59)	
Consult with physician	18 (30.5)
Sputum smear	9 (15.3)
Sputum smear + Chest X-Ray	10 (16.9)
Attributed to underlying disease	10 (16.9)
Self-medicated	7 (11.9)
None	5 (8.5)

Values are *n* (%)

*TST* Tuberculin skin test, *IPT* Isoniazid preventive therapy

Overall, 56.5% of participants (95% CI: 49.2–63.5%) had a positive TST result. The mean size of the induration was 17.2 mm (SD = 5.1) and 1.6 mm (SD = 2.8) when the TST results were positive and negative, respectively. The prevalence of a positive TST result among HCWs was 64.3% (95% CI: 57.8–70.3%) when including participants with previous positive TST results or a history of TB. Table 4 shows that greater time working as a HCW (95%CI 146.10–186.86) and greater time working in the health center (95%CI 97.28–139.88) were associated with a positive TST result. Older age was also associated with a positive TST result (95% CI 41.55–44.88). No significant differences were observed between clinical/paramedical and administrative staff in the health center.

**Table 4:** Comparison between TST positive and negative groups

Variables	TST negative (***n*** = 80)	TST positive (***n*** = 144)	PR	***P*** value
**Gender**				
Female	68 (85)	117 (81.2)	1.09	0.45
**District of residency**				
SJL	45 (56.2)	81 (56.2)	1.00	1.0
**Use of N95 mask**				
Yes	44 (55)	85 (59)	0.88	0.3
**TB household contact**				
Yes	17 (21.2)	32 (22.2)	1.18	0.22
**Cough > 15 days during the last year**				
Yes	18 (22.5)	36 (25)	1.05	0.43
**BMI**				
< 25	32 (40)	58 (40.3)	0.99	0.97
≥ 25	48 (60)	86 (59.7)	Ref.	
**Occupation**				
Clinical/paramedical staff	48 (60)	88 (61.1)	0.86	0.25
Administrative	17 (21.2)	32 (22.2)	Ref.	
**Age, years, median (SD)**	38.3 (12.47)	43.2 (10.11)	1.01	0.004
**Time working as health worker, months, mean (SD)**	118.2 (122.2)	166.5 (123.7)	1.00	0.005
**Time working on health center, months, mean (SD)**	73.7 (99.9)	118.6 (129.4)	1.00	0.001

Values are n (%) unless noted otherwise

*TST* Tuberculin skin test, *PR* Prevalence ratios, *SJL* San Juan de Lurigancho, *TB* Tuberculosis, *BMI* Body mass index

*TST negative* Participants with negative TST result, *TST positive* Participants with positive TST, previous positive TST and history of TB

Chi-squared test used for categorical variables. Student’s t-test used when means are displayed

In the bivariate analysis, the PRs show that workers with more than 120 months as a HCW were 1.44 times more likely to be TST positive. Being overweight, having reported a household TB contact, and not using N95 masks were not significantly associated with a positive TST result. In the multivariate analysis, HCWs with over 10 years of experience were 1.52 times more likely to be TST positive when compared to the factors used in the bivariate analysis that, albeit not statistically significant, we consider to be important determinant factors for a TST positive result (Table 5). Age and time working in the health center were not included in the univariate or multivariate analysis since they are closely related to time working as a HCW, which is a more specific measure for TB exposure.

**Table 5:** Factors associated with TST positive result

Variables	PR (Bivariate analysis)	95% CI	PR (Multivariate analysis)	95% CI	P value
**Overweight (BMI > 25)**	0.99	0.81–1.21	0.99	0.80–1.23	0.94
**Household TB contacts**	1.18	0.90–1.56	1.02	0.73–1.42	0.90
**Not using N95 mask**	1.13	0.89–1.45	1.08	0.85–1.38	0.54
**Time working as HCW (> 120 months)**	1.44	1.16–1.77	1.52	1.19–1.95	0.001

*PR* Prevalence ratio, *BMI* Body Mass Index, *TB* Tuberculosis, *HCW* Healthcare worker

## Discussion

Our study documented that 56.5% of the 184 health workers had a positive TST result; this prevalence rose to 64.3% when we included workers with previous positive TST results or a history of active TB disease. Both estimates are within the range expected for HCWs in low-income countries (33%) and middle-income countries (79%) [13]. Recent systematic reviews have found a prevalence of 49 and 37% with a mean active TB incidence rate of 97 new cases per 100,000 HCWs [7, 8]. In addition, our result is slightly greater than the prevalence of 56% reported in a study among HCWs in Callao using IGRA [10]. These rates depict HCWs as a population at risk of developing active TB, since more than half of them have LTBI.

Similar to our study, *Soto-Cabezas* et al. in Peru found a significant association between LTBI and both age and time working as a HCW [10]. Likewise, *Rafiza* et al. in Malaysia uncovered an increased prevalence of LTBI in employees with more than 11 years of work (OR: 3.48), and *Pai* et al. in India reported an association between LTBI and 10 or more years on the job, a three-fold increase in prevalence compared to those employed for less than 1 year [16, 17]. An active TB patient with smear-positive sputum will infect, on average, between 10 to 15 people every year [18]; since most people will go to health care centers as their first point of contact for diagnosis, treatment, and monitoring of TB, it is not surprising that a longer time of employment in this health care setting reflects repetitive exposure to *M. tuberculosis*. Such repetitive exposure is concerning since it has not only been reported that individuals have a 10% chance of progression to active disease after a single TB exposure, but also that the probability of progression is greater among individuals with 18 or more exposures [19].

Although time spent with TB patients has been shown to be a risk factor for LTBI [7, 13, 20, 21], our analyses suggested that clinical and laboratory staff were not at higher risk for LTBI than administrative workers. This lack of association between TST results and occupation contradicts previously reported findings [7]. However, the inconsistency can be explained by the fact that health centers in SJL are limited in space, so common waiting areas and rooms are in close proximity to the area assigned for daily TB treatment. Thus, levels of TB exposure may be similar for all staff, unlike in other settings such as large hospitals. It is also possible that the lack of association between TST results and occupation may be due to the staff’s exposure to either the high prevalence of TB outside of the health center or a household contact. Moreover, the observed TST positivity may have been due to TB infection during childhood or the Bacille Calmette-Guérin (BCG) vaccination given universally in Peru at birth. Most of our participants were women, which is often the case in the healthcare setting since women constitute more than half of the national healthcare workforce [12]. We found no association between gender and TST result.

The implementation of TB transmission control measures such as natural ventilation, a supply of N95 masks, and routine screening, is essential to protect HCWs and may decrease annual TB incidence by as much as 49, 27, and 81% in countries with low, intermediate, and high TB incidence, respectively [22]. Previously reported obstacles preventing the use of N95 masks identified by HCWs are heat around the face and inaccessibility to the masks [23]. Our study found that 60.3% of HCWs did not wear N95 masks because the health center failed to provide them. This inaccessibility to personal protective equipment is a flaw in TB control which must be avoided in all settings, especially in countries with high TB incidence. Nonetheless, HCWs usually only wear masks when dealing with known TB cases. Since it is not common practice to continuously wear masks, TB transmission in healthcare facilities may be due to undiagnosed cases [24].

Only half of our participants had had previous active TB screening during the last year. Though it is standard procedure for health care centers to perform these screenings annually, this study reveals that health care centers are unable to ensure TB screening for all of their workers. Furthermore, considering the high LTBI prevalence uncovered in our study, it seems unlikely that only 26 participants were exempt from TST application due to a previous positive TST result. Rather, it is more likely that HCWs overreported having been tested in the last year, instead of underreporting a positive TST. In Portugal, from a sample size of 2015 registered physicians and nurses, a survey reported that 784 (39.5%) were never screened and, of those HCWs, 741 (94.5%) were never offered screening [25]. Moreover, in China, where policies on medical TB surveillance among HCWs have not been implemented, a large study identified 124 HCWs with presumptive active TB, while noting that the screening methods and framework used were not yet optimal for the high-burden of TB in the country [26]. Accordingly, either routine or post-exposure screening procedures for HCWs should be established to ensure their health.

Even though 85.2% of participants agreed to take IPT if their result was positive, some refused the prophylaxis due to misinformation about the risk of possible adverse events and immunological reactions or belief in their own pre-existing immunity against TB. Although standards of TB care in Peru dictate that IPT must be administered to health workers and prison staff within 2 years of a positive TST result after ruling out active TB [27], studies found acceptance rates for chemoprophylaxis amongst HCWs between 65 and 84% in low TB prevalence settings [28, 29]. However, the use of IPT is debatable in settings with a high prevalence of MDR-TB, as in Peru [1], which could explain why HCWs refuse the prophylaxis and prefer close monitoring of TB symptoms for at least 2 years, as per WHO recommendations [3]. Nevertheless, education and close monitoring of active TB symptoms must be provided for all HCWs, not only those who are TST positive.

Finally, it is important to address this study’s limitations. Recall bias may have affected the participants’ responses to questions about previous indentation, TST results, or whether or not they had presented with a confirmed case of active TB. An important possible confounding factor is that we were not able to obtain participants’ full life histories prior to beginning work in the healthcare system; thus, exposure to TB could have occurred before working as a HCW, especially if they lived in SJL or another high TB burden area in Lima. Along the same lines, we cannot be sure that we received reliable information on BCG vaccination. Self-reported use of N95 masks is also not reliable, as HCWs may feel compelled to overreport its use; additionally, the survey did not ask for a quantitative measure of mask use frequency, so this variable was open to interpretation by the HCWs completing the questionnaire. Finally, the power of the study may have been lower than necessary to detect relationships between other important variables that were not statistically significant in the multivariable analysis.

## Conclusion

This study supports previous reports that TB infection is an occupational hazard for HCWs. Although we were not able to identify specific areas in the health center where workers are more likely to be exposed, measures of TB control should be universally instituted throughout health centers, especially considering the potential these measures have to reduce TB incidence. The high prevalence of LTBI and its associated risk for active disease emphasizes the need for regular screening in addition to adequate TB control measures. Prevention of active TB, as well as timely diagnosis of LTBI in HCWs, is critical for ensuring individual and public health.

## Supplementary information

**Additional file 1.** Questionnaire (English version).

## Data Availability

All data generated or analyzed during this study is available upon reasonable request to the corresponding author.

## Abbreviations

BCG: Bacille Calmette-Guérin
CI: Confidence interval
HCW: Healthcare workers
HIV: Human immunodeficiency virus
IGRA: Interferon-gamma release assays
IPT: Isoniazid preventive therapy
IQR: Interquartile range
LTBI: Latent tuberculosis infection
MDR: Multidrug-resistant
OR: Odds ratio
PR: Prevalence ratio
SD: Standard deviation
SJL: San Juan de Lurigancho
TB: Tuberculosis
TST: Tuberculin skin test
WHO: World Health Organization
