# Supplementary material for: Prevalence of positive TST among healthcare workers in high-burden TB setting in Peru
Source: BMC Public Health. 2020 May 3;20:612. doi: 10.1186/s12889-020-08756-9 (PMC7197122; doi:10.1186/s12889-020-08756-9)
Supplement: Supplementary file 1 — Additional file 1. Questionnaire (English version). [file 12889_2020_8756_MOESM1_ESM.docx]

SECTION 1: STUDY IDENTIFICATION

| Date of enrollment | ___ / ___ / ___  day month year |
| --- | --- |
| Health center | _______________________________________ |
| Participant code | \|  \|  \|  \|  \|  \| \| --- \| --- \| --- \| --- \| --- \| |

SECTION 2: CONTACT INFORMATION

| NAME | _____________________ _____________________  First name. Middle name |
| --- | --- |
|  | _____________________ ____________________  Last name (Father) Last name (Mother) |
| GENDER | Female = 0 Male = 1 |
| DATE OF BIRTH | __ __ / __ __ / __ __ __ __  day month year |
| PLACE OF BIRTH | ____________________________________________ |
| AGE | \|  \|  \| \| --- \| --- \| |
| ADDRESS | ___________________________________________________________ |
| DISTRITO | SJL = 1 Other = 2 ____________________________ |
| TIME LIVING IN DISTRICT |  |
| TELEPHONE NUMBER | __ __ __ __ __ __ __ __ __ __ __ __ __ __ __ __ __ __ |
| NATIONAL ID NUMBER | \|  \|  \|  \|  \|  \|  \|  \|  \| \| --- \| --- \| --- \| --- \| --- \| --- \| --- \| --- \| |
| EMERGENCY CONTACT | 1 = Si 2 = No |
| NAME OF EMERGENCY CONTACT | _____________________________________________ |
| TELEPHONE OF EMERG. CONTACT | __ __ __ __ __ __ __ __ __ __ __ __ __ __ __ __ __ __ |

SECTION 3. QUESTIONNAIRE

| Q1 | Did you receive BCG vaccination? | NO (0) | YES (1) |
| --- | --- | --- | --- |
| Q2 | Occupation in health center  ⃝ Physician =1 ⃝ Obstetrician =4 ⃝ Lab technician =7  ⃝ Nurse =2 ⃝ Health promoter =5 ⃝ Nurse technician =8  ⃝ Psychologist =3 ⃝ Nutricionist =6 ⃝ Social worker = 9  ⃝ Other =10 __________________ |  |  |
| Q3 | Time working in health center : ______________ (months/years) |  |  |
| Q4 | In the previous year, did you have contact with a TB patient? | NO (0) | YES (1) |
| Q5 | Did you use a N95 mask when in contact with a TB patient? | NO (0) | YES (1) |
|  | If the answer was yes  ⃝ Sometimes=1 ⃝ Almost always=2 ⃝ Always=3 |  |  |
| Q7 | If the answer to the previous question was yes, please answer:  Was this a household TB contact? | NO (0) | YES (1) |
| Q8 | Have you received isoniacid preventive therapy (IPT)? | NO (0) | YES (1) |
| Q9 | If the answer to Q10 was yes, please answer:  For how long? : ________________ (months) |  |  |
| Q10 | Have you ever been diagnosed with active TB? | NO (0) | YES (1) |
|  | If the answer was yes: In which year? ________________  (If there was more than one episode, please choose the latest) |  |  |
|  | If the answer to Q10 was yes, did you receive treatment? | NO (0) | YES (1) |
|  | If the answer to the previous question was yes, what treatment did you receive? _______________________________________________________________ |  |  |
|  | Duration of treatment? ________________________ |  |  |
| Q11 | In the previous year, did you have active TB screening with a sputum smear? | NO (0) | YES (1) |
|  | What was the result? _________________ |  |  |
| Q12 | In the previous year, did you have active TB screening with a chest X-ray? | NO (0) | YES (1) |
|  | What was the result? _________________ |  |  |
| Q13 | In the previous year, did you have latent TB screening with a TST? | NO (0) | YES (1) |
|  | What was the measured PPD induration? _________________ cm |  |  |
| Q14 | Would you take IPT is the TST administered reports as positive? | NO(0) | YES (1) |
| Q15 | ¿Le han diagnosticado a usted alguna de estas enfermedades?  ⃝ HIV =1 ⃝ Diabetes Mellitus=3 ⃝ Hipertension=4  ⃝ Chronic kidney disease=2 ⃝ Others=5_________________ |  |  |
